# Coupling Aggressive Mass Removal with Microbial Reductive Dechlorination for Remediation of DNAPL Source Zones: A Review and Assessment

**DOI:** 10.1289/ehp.6932

**Published:** 2004-12-08

**Authors:** John A. Christ, C. Andrew Ramsburg, Linda M. Abriola, Kurt D. Pennell, Frank E. Löffler

**Affiliations:** ^1^Environmental and Water Resources Engineering Program, University of Michigan, Ann Arbor, Michigan, USA; ^2^Department of Civil and Environmental Engineering, Tufts University, Medford, Massachusetts, USA; ^3^School of Civil and Environmental Engineering, Georgia Institute of Technology, Atlanta, Georgia, USA

**Keywords:** aquifer, bioremediation, DNAPL, innovative technologies, microbial degradation, physical–chemical, post-treatment, reductive dechlorination, remediation, source zone

## Abstract

The infiltration of dense non-aqueous-phase liquids (DNAPLs) into the saturated subsurface typically produces a highly contaminated zone that serves as a long-term source of dissolved-phase groundwater contamination. Applications of aggressive physical–chemical technologies to such source zones may remove > 90% of the contaminant mass under favorable conditions. The remaining contaminant mass, however, can create a rebounding of aqueous-phase concentrations within the treated zone. Stimulation of microbial reductive dechlorination within the source zone after aggressive mass removal has recently been proposed as a promising staged-treatment remediation technology for transforming the remaining contaminant mass. This article reviews available laboratory and field evidence that supports the development of a treatment strategy that combines aggressive source-zone removal technologies with subsequent promotion of sustained microbial reductive dechlorination. Physical–chemical source-zone treatment technologies compatible with posttreatment stimulation of microbial activity are identified, and studies examining the requirements and controls (i.e., limits) of reductive dechlorination of chlorinated ethenes are investigated. Illustrative calculations are presented to explore the potential effects of source-zone management alternatives. Results suggest that, for the favorable conditions assumed in these calculations (i.e., statistical homogeneity of aquifer properties, known source-zone DNAPL distribution, and successful bioenhancement in the source zone), source longevity may be reduced by as much as an order of magnitude when physical–chemical source-zone treatment is coupled with reductive dechlorination.

Widespread use of chlorinated solvents in dry cleaning and metal degreasing operations over the last century has resulted in extensive groundwater contamination by compounds such as tetrachloroethene (PCE) and trichloroethene (TCE). When released into the subsurface as dense non-aqueous-phase liquids (DNAPLs), chlorinated solvents tend to migrate downward through the unsaturated zone and can penetrate the water table because of their higher density (Mercer and Cohen 1990). During DNAPL migration, hysteretic capillary forces cause retention of a portion of the liquid within the pores as discontinuous globules or ganglia [Lenhard et al. 1989; Schwille 1988; U.S. Environmental Protection Agency (U.S. EPA) 1990]. Substantial DNAPL volumes can also be retained because of the presence of nonuniform soil texture, which may result in DNAPL pooling (i.e., zones of DNAPL at much higher saturation) above layers or lenses of lower-permeability media (Dekker and Abriola 2000; Essaid and Hess 1993; Saenton et al. 2002; Schwille 1988). The resulting distribution of DNAPL is, thus, typically complex and nonuniform (Figure 1). Entrapped DNAPL mass tends to dissolve slowly into flowing water, serving as a long-term source of groundwater contamination (Mackay and Cherry 1989; Schwille 1988). The implementation of conventional pump-and-treat remediation for such DNAPL source zones has been ineffective in reducing contaminant concentrations to regulatory end points in acceptable time frames (MacDonald and Kavanaugh 1994; Travis and Doty 1990; U.S. EPA 1996).

A number of innovative technologies have been developed to enhance contaminant removal from DNAPL source zones [National Research Council (NRC) 1994, 1997, 1999). Although these technologies are capable of substantial mass removal under favorable conditions, some DNAPL will likely remain within the porous medium even when treatment is most effective (Fountain et al. 1995; Sale and McWhorter 2001). This remaining contaminant mass can continue to serve as a source of down-gradient contamination, and thus further source-zone treatment or containment may be required. Despite a number of successful field-scale demonstrations of aggressive source-zone treatment technologies, skepticism and concern remain that application of such technologies may not substantially reduce risk and could potentially worsen site conditions (e.g., through mobilization and redistribution of DNAPL, enhanced transport of metals, elimination of microbial activity, or increased aqueous-phase concentration of contaminants after treatment) (Cherry et al. 1997; Oostrom et al. 1999). From this perspective, some authors have suggested that source containment (i.e., treatment or mitigation of down-gradient contamination emanating from DNAPL source zones) is preferable to aggressive physical–chemical source-zone treatment (Cherry et al. 1997; Freeze 2000; Freeze and McWhorter 1997; Kent and Mosquera 2001).

Freeze (2000) advocates a new remediation paradigm in which only source containment is implemented because of the technical impracticability of removing sufficient DNAPL mass to reduce contaminant concentrations to drinking water standards. In contrast, guidelines put forth by the Interstate Technology and Regulatory Cooperation work group, a team composed of state and federal regulators, call for aggressive source-zone remediation (Jackson 2001). The latter recommendation is based in part on the contention that mass removal from a source zone, even if incomplete, will result in *a*) a reduction in mass flux, *b*) a reduction in source longevity, *c*) a reduction in risk, and *d*) a potential enhancement in posttreatment biodegradation (Jawitz et al. 2000; Londergan et al. 2001; Martel et al. 1998; Rao et al. 2002; Yang and McCarty 2003). Recent analytical and numerical modeling investigations suggest that partial source-zone removal may result in significant (several orders of magnitude) reductions in posttreatment contaminant mass flux (Lemke and Abriola 2003; Rao et al. 2002; Rao and Jawitz 2003). Although a reduction in mass flux may not eliminate the need for further treatment, it could reduce concentrations to levels where microbial transformation of the dissolved-phase chlorinated solvents becomes feasible (Adamson et al. 2003; Nielsen and Keasling 1999; Sung et al. 2003; Yang and McCarty 2000). Biostimulation of source-zone microbial dechlorination activity may achieve attenuation of contaminant mass flux to levels that achieve regulatory compliance (i.e., a flux averaged concentration) at a down-gradient well.

Thus, combination of physical–chemical source-zone treatment and posttreatment bioremediation may be an attractive remediation alternative, resulting in reduced source longevity and contaminant mass flux (de Blanc et al. 1997; Rao et al. 2002; Zoller 1998; Zoller and Rubin 2001). Coupling a physical–chemical remediation process that removes significant contaminant mass with a bioremediation “polishing step” to control the contaminant mass flux emanating from remaining DNAPL may provide a synergism that cannot be obtained with existing remediation strategies. Such a staged treatment approach could leverage initial high removal efficiencies of physical–chemical source-zone treatment methods to minimize time to site closure. This sequential treatment approach should not be confused with natural attenuation, a remediation approach generally associated with bioremediation of low contaminant concentrations in a groundwater plume (Wiedemeier et al. 1999), nor should it be confused with the recent work on source-zone bioremediation, which relies solely on biotic processes to transform source-zone contamination (e.g., Adamson et al. 2003).

Observations from longer term monitoring at sites where innovative flushing technologies have been implemented suggest that tailoring physical–chemical treatment to enhance post-treatment bioremediation efforts is feasible (Mravik et al. 2003; Ramsburg et al. 2004). Application of such a staged treatment methodology, however, would require a thorough understanding of both physical–chemical treatment technologies and source-zone bio-remediation. Our objective in this article is to review and integrate knowledge gained from recent demonstrations of field-scale source-zone remediation with that from laboratory investigations of solvent biotransformation to assess the potential promise of technology coupling. This work differs from published reviews of specific technologies (e.g., Bradley 2003; Henry et al. 2003) in its focus on the influence of physical–chemical treatment technologies on posttreatment microbial reductive dechlorination. A technology assessment is provided and recommendations for future work are presented. Although some observations may be generally applicable to any DNAPL site, the focus herein is on sites where source-zone contamination mainly comprises chlorinated solvents (e.g., PCE, TCE).

## Chlorinated Ethene Biodegradation

The degradation of chlorinated ethenes in microcosms and the detection of degradation products at contaminated groundwater sites in the 1980s inspired researchers to investigate biotic and abiotic transformation processes (McCarty and Semprini 1994; Vogel and McCarty 1985; Vogel et al. 1987). As early as 1980, researchers identified links between microbial metabolism and the destruction of chlorinated hydrocarbons (Higgins et al. 1980). As more work was completed, researchers recognized that oxidation or reduction of chlorinated hydrocarbons under different redox conditions is feasible (Table 1). The following discussion briefly reviews microbial dechlorination processes that can occur in the subsurface and identifies those processes that are most promising for stimulation in a source zone after active mass removal. For a more thorough discussion and review of chloroethene biodegradation, see Bradley (2003), Holliger (1995), Janssen et al. (2001), Semprini (1997, 2001), or Smidt and de Vos (2004).

Although oxidation of chlorinated hydrocarbons in both aerobic and anaerobic environments has been demonstrated (Bradley et al. 1998; Bradley and Chappelle 1996; Coleman et al. 2002a, 2002b; Hartmans et al. 1985; Hartmans and deBont 1992; Singh et al. 2004; Verce et al. 2000, 2001), aerobic metabolic oxidation is a productive pathway only for removal of lesser chlorinated ethenes [i.e., *cis*-dichloroethene (*cis*-DCE) and vinyl chloride (VC)]. No organisms that grow aerobically with PCE or TCE as a carbon source have been identified. In anoxic environments the metabolic oxidation of chloroethenes is still poorly understood. Although the mineralization of *cis*-DCE and VC under iron- and manganese-reducing conditions has been demonstrated (Bradley et al. 1998; Bradley and Chapelle 1996), the relevance of this process for bioremediation has yet to be established. Co-metabolism is an alternative nonmetabolic process that has been shown to transform contaminants in both aerobic and anaerobic environments (Anderson and McCarty 1997; Chauhan et al. 1998; Ensign et al. 1992; Hopkins et al. 1993; Ryoo et al. 2001; Shim et al. 2001). Aerobic co-metabolism can act on all chloroethenes (Ryoo et al. 2001; Shim et al. 2001); however, the need for a primary substrate such as methane or toluene, and the fact that the degradation of the target compounds can only be indirectly controlled are major drawbacks of this approach. Anaerobic co-metabolic reductive dechlorination of PCE has been observed under methanogenic (Fathepure and Boyd 1988a, 1988b), acetogenic (Terzenbach and Blaut 1994), and sulfidogenic conditions (Cole et al. 1995). However, because of low rates and incomplete dechlorination, this process is least likely to contribute to detoxification of contaminated subsurface environments. More recently, chlororespiration, a process in which chlorinated compounds serve as a metabolic electron acceptor for energy generation, has been demonstrated (Holliger et al. 1998; Löffler et al. 1996, 1999; Smidt and de Vos 2004). The metabolic reductive dechlorination pathway (chlororespiration) is a strict anaerobic process that requires an electron donor (i.e., source of reducing equivalents). The chloro-respiratory pathway is promising in that it can lead to efficient dechlorination to ethene and achieve complete detoxification (He et al. 2003a, 2003b).

The ability to use chloroethenes as energy-yielding electron acceptors is distributed among several bacterial groups, including different subdivisions of the proteobacteria, the gram-positive bacteria, and the *Chloroflexi* (formerly green nonsulfur bacteria). Organisms capable of metabolic reductive dechlorination (i.e., chlororespiration) have been isolated from contaminated and pristine sites (Smidt and de Vos 2004). These populations are generally strict anaerobes, with only *Enterobacter* strain MS-1 exhibiting facultative metabolism (Sharma and McCarty 1996). Bacterial populations capable of gaining energy from reductive dechlorination of chloroethenes have been classified into a number of phylogenetic groups, including *Dehalobacter, Sulfurospirillum, Desulfuromonas, Desulfitobacterium, Clostridium*, and *Dehalococcoides* (Bradley 2003; Löffler et al. 2003; Smidt and de Vos 2004). This broad range of organisms capable of chlororespiration is encouraging for posttreatment bioremediation; however, most of these organisms are incapable of complete dechlorination of chloroethenes to ethene (Löffler et al. 2003; Major et al. 2003). At many sites, DCEs (primarily *cis*-DCE) and, in some cases, VC accumulate. Cupples et al. (2004) recently demonstrated dechlorination of *cis*-DCE and VC, but they identified a minimum threshold chlorinated contaminant concentration below which dechlorination could not be sustained. There is an apparent link between the presence of members of the *Dehalococcoides* group and complete dechlorination (i.e., ethene formation) (Cupples et al. 2003; He et al. 2003a, 2003b; Hendrickson et al. 2002; Maymo-Gatell et al. 1997, 2001; Ritalahti et al. 2001). *Dehalococcoides ethenogenes* strain 195 was the first isolate described to dechlorinate PCE to ethene, but the last dechlorination step, VC to ethene, was co-metabolic and slow (Maymo-Gatell et al. 1997). A major breakthrough was the isolation of *Dehalococcoides* species strain BAV1, the first isolate capable of using all DCE isomers and VC as growth-supporting electron acceptors (He et al. 2003a, 2003b).

Although it was originally believed bio-transformation processes could not occur near a chlorinated solvent source zone because of the toxicity of high contaminant concentrations associated with the presence of NAPL (Abelson 1990; Bouwer 1994; Robertson and Alexander 1996), recent chlororespiration investigations have been performed in the presence of non-aqueous-phase PCE (Adamson et al. 2004; Carr et al. 2000; Cope and Hughes 2001; Dennis et al. 2003; Nielsen and Keasling 1999; Sung et al. 2003; Yang and McCarty 2000, 2002). Nielsen and Keasling (1999) demonstrated complete reductive dechlorination (e.g., ethene formation) at saturated PCE concentrations in batch systems with a dechlorinating consortium. Most reducing equivalents from the electron donor (glucose) were consumed in reductive dechlorination, probably due to the inhibition of other microbial processes by the high chloroethene concentrations. Yang and McCarty (2000) also reported degradation of PCE in batch systems where concentrations of PCE approached the aqueous solubility limit. Although dechlorination stalled at *cis*-DCE, incomplete dechlorination could still be beneficial for source-zone bioremediation because *a*) dissolution rates are enhanced 3-fold (Yang and McCarty 2002, 2003) to 6-fold (Cope and Hughes 2001) and *b*) *cis*-DCE and VC are more accessible to aerobic degradation in down-gradient aerobic zones (Coleman et al. 2002a, 2002b). In column studies, a nonuniform distribution of NAPL and organisms resulted in significant competition for reducing equivalents and bio-clogging due to excessive microbial growth of nondechlorinating biomass (Yang and McCarty 2002). Competition and bioclogging may be controlled by slow-release electron donors. However, application of a simplified numerical model suggested that under electron-donor–limiting conditions, a biofilm develops around the NAPL, reducing dissolution and increasing the difficulty of supplying sufficient electron donor (Chu et al. 2003). Partitioning of lesser chlorinated ethenes (TCE, *cis*-DCE, VC) into PCE-DNAPL and decreases in pH due to the release of HCl have also been observed and may affect the dechlorination of the lesser chlorinated ethenes (Adamson et al. 2004; Cope and Hughes 2001).

These findings have important ramifications for source-zone bioremediation, as well as posttreatment biopolishing. Although a variety of organisms are capable of PCE–to–*cis*-DCE dechlorination, complete detoxification requires the presence and activity of *Dehalococcoides* populations (Hendrickson et al. 2002; Ritalahti et al. 2001). Contaminant removal and plume containment after bioaugmentation with *Dehalococcoides*-containing cultures have been demonstrated in the field (Ellis et al. 2000; Lendvay et al. 2003; Major et al. 2002), and recent results suggest that bioaugmentation is also a viable approach for initiation of reductive dechlorination in PCE source zones (Adamson et al. 2003). These findings suggest that combined bioaugmentation strategies that *a*) initiate the reductive dechlorination process in source zones (Adamson et al. 2003) after physical–chemical treatment and *b*) establish bioreactive barriers for treatment of dissolved contaminants down-gradient (Lendvay et al. 2003) are promising remediation approaches that warrant further exploration.

To sustain the reductive dechlorination process, a source of reducing equivalents (i.e., an electron donor) must be provided. Chlororespiring populations depend on the activity of fermentative organisms to convert (complex) organic materials into suitable electron donors (e.g., hydrogen or acetate) (DiStefano et al. 1992; He et al. 2002). A variety of substrates including pentanol, ethanol, lactate, propionate, butyrate, and oleate have been shown to produce suitable electron donors (e.g., acetate, hydrogen) to support chlororespiring populations (Carr and Hughes 1998; Fennell and Gossett 1998; He et al. 2002; Yang and McCarty 1998, 2002). Alternative amendment strategies that supply slow-release, nonsoluble substrates for example, olive oil, chitin, polylactate esters [e.g., Hydrogen Release Compound (HRC; Regenesis Bioremediation Products, San Clemente, CA)], have also been successfully, used (Koenigsberg and Farone 1999; Yang and MacCarty 2002). Chlororespiring populations are highly competitive hydrogen users and outcompete methanogens, acetogens, and sulfate-reducing populations for this electron donor (Löffler et al. 1999). Thus, substrates that result in slow release (or production) of hydrogen are advantageous because most reducing equivalents are directed toward the process of interest (Ballapragada et al. 1997; Fennell et al. 1997; Fennell and Gossett 1998; He et al. 2002; Smatlak et al. 1996). It should be noted that any approach that increases the flux of hydrogen in a subsurface environment will also result in an increased flux of acetate, which has been implicated as a relevant source of low concentrations of hydrogen through syntrophic oxidation (He et al. 2002; Schink 1997).

## Physical–Chemical Treatment of Chlorinated Solvent Source Zones

Over the past decade, a number of innovative technologies have been developed that show promise for recovering a large fraction of the DNAPL mass at a given site (e.g., Brusseau et al. 1999; Stroo et al. 2003). Although the number of field-scale demonstrations of these technologies is growing, more standardization of assessment and reporting of results are necessary before larger-scale implementations can be considered sound practice (NRC 1997). Furthermore, the lack of consensus pertaining to the potential benefits of partial source-zone removal (e.g., Rao et al. 2002; Rao and Jawitz 2003; Sale and McWhorter 2001) points to the need for a better understanding of the long-term influence of physical–chemical treatment on contaminant fluxes, plume development, and enhanced microbial activity.

Given that innovative source-zone removal technologies have been extensively documented (e.g., NRC 1994, 1997, 1999), this article provides only a brief summary of selected approaches including air sparging, chemical oxidation, thermal treatment, co-solvent flushing, and surfactant-enhanced aquifer remediation (SEAR). Application of any of these treatment technologies would require detailed site characterization, a well-delineated source zone, and, in most cases, efficient contact between injected fluids and DNAPL. The discussion below focuses on assessing the potential for coupling each technology with microbial reductive dechlorination.

### Air sparging.

**Figure 2:**
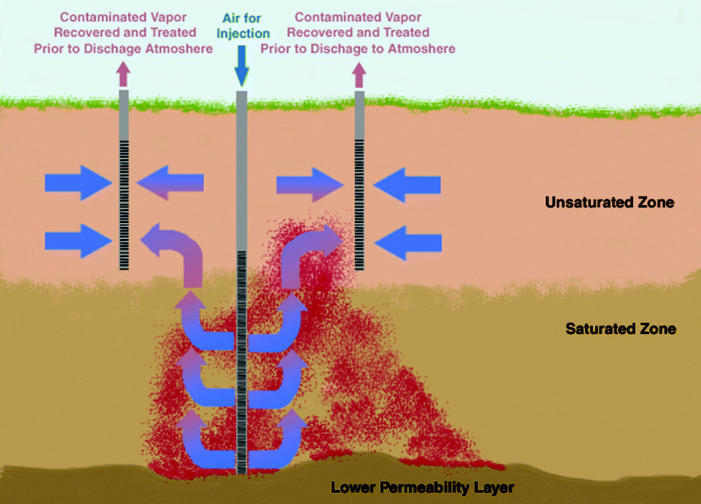
Representation of air sparging with soil vapor extraction in a shallow, relatively homogeneous, unconfined aquifer with a well-defined DNAPL source zone. Arrows represent tortuous air channels into which contaminants partition and are subsequently recovered through soil vapor extraction wells.

A source-zone remediation technology that has been implemented at many DNAPL-contaminated sites is air sparging (NRC 1997; for more detailed descriptions and reviews of air sparging technologies, see Brown 1997; Hinchee 1994; Johnson et al. 1993; Reddy et al. 1995; Suthersan 1996). Air is injected below the water table to volatilize or strip contaminants from groundwater (Figure 2). The vapor-phase contaminant rises into the unsaturated zone, where it can then be extracted with a soil vapor extraction system (Johnson et al. 1993). Typically, design of these systems is empirical and based upon two primary assumptions: *a*) the gas phase will contact the nonaqueous phase, resulting in direct mass transfer from the DNAPL to the vapor phase, and *b*) the gas phase will strip dissolved contaminants from the aqueous phase (Suthersan 1997; Unger et al. 1995).

Although air sparging may be applied to reduce DNAPL mass (Unger et al. 1995), concerns remain that the introduction of air to a source zone may increase the extent of contamination through lateral and vertical spreading of NAPL (Blanford et al. 1999; Henry et al. 2003). Air sparging has been reported to stimulate aerobic microbial processes, including co-metabolism of chlorinated ethenes, as long as a suitable primary substrate is present (Gierke et al. 1999; Johnson et al. 1993; Raes et al. 2002). Sustained enhanced aerobic biodegradation, however, may be problematic because aerobic degradation of unsaturated chlorinated solvents is limited at the high contaminant concentrations commonly found within DNAPL source zones (Alvarez-Cohen and McCarty 1991). The implementation of the aerobic co-metabolic process has been successfully demonstrated for TCE removal under field conditions (McCarty et al. 1998); however, the requirement for a primary substrate (e.g., toluene) remains problematic. Although lower-chlorinated ethenes (e.g., *cis*-DCE and VC) are amenable to growth-linked microbial degradation under aerobic conditions, a metabolic process capable of oxidizing PCE and TCE has yet to be identified (Löffler et al. 2003). For these reasons, it is unlikely that stimulation of reductive dechlorination after air sparging is a viable approach.

### Chemical oxidation.

*In situ* chemical oxidation (ISCO) was developed to transform contaminants into benign products (i.e., CO_2_ and salts) [for mechanistic descriptions of ISCO technologies, see NRC (1999) and Siegrist et al. 2001]. A common form of this technology involves the injection of hydrogen peroxide (~10 to 50% by weight) in conjunction with an iron catalyst (e.g., ferrous sulfate), which forms highly reactive hydroxyl radicals (OH^•^) via Fenton’s chemistry. The hydroxyl radicals are strong oxidants and react rapidly with surrounding molecules. Solutions of hydrogen peroxide, without catalyst, have been introduced into the subsurface (Oberle and Schroder 2000) to reduce iron catalyst requirements and the need for pH adjustments. However, hydrogen peroxide at ambient temperature and pressure is a relatively poor oxidizing agent for chlorinated solvents. When hydrogen peroxide solutions are injected alone (i.e., without an iron catalyst), reductions in contaminant concentrations are frequently the result of volatilization or stripping, which occurs because of increased temperature and O_2_ production as the hydrogen peroxide decomposes (Oberle and Schroder 2000). Permanganate, in the form of either sodium permanganate or potassium permanganate, offers an attractive alternative to Fenton’s chemistry because it does not rely on the formation and transport of short-lived OH^•^ radicals. The use of permanganate, however, results in the formation of manganese dioxide, which may precipitate and reduce aquifer permeability (Dai and Reitsma 2002; Li and Schwartz 2003; Siegrist et al. 2001). The potential for permeability reduction, as well as increased metal mobility, that may accompany use of chemical oxidants depends upon site-specific geochemical conditions. Thus, as with all source-zone treatment technologies, thorough site characterization is required to mitigate potential adverse effects (Crimi and Siegrist 2003; Siegrist et al. 2001).

**Figure 3:**
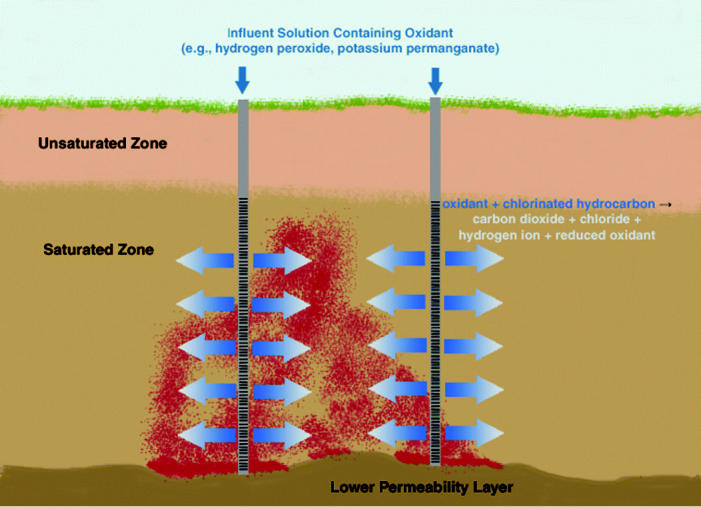
Representation of ISCO in a shallow, relatively homogeneous, unconfined aquifer with a well-defined DNAPL source zone. Contaminant destruction occurs *in situ* as depicted by the representative chemical reaction. Alternatively, implementation of ISCO technologies may use a point-to-point flood similar to that shown in Figure 4.

Application of chemical oxidation to DNAPL source zones (Figure 3) has produced mixed results (Siegrist et al. 2001; Urynowicz and Siegrist 2000). Still, some evidence suggests that permanganate oxidation of DNAPLs may be plausible if delivery of chemical oxidants to DNAPL mass can be improved (Nelson et al. 2001; Schnarr et al. 1998; West et al. 1998) and MnO_2_ crusting of the DNAPL avoided (Dai and Reitsma 2002; Li and Schwartz 2003; Siegrist et al. 2001). These issues notwithstanding, the fate of microorganisms through the oxidation process remains unclear (Bassel and Nelson 2000; Kastner et al. 2000). Although a limited number of studies indicate that both aerobic and anaerobic populations may rebound after treatment with relatively low concentrations (< 2% weight) of oxidants (e.g., Allen and Reardon 2000), the posttreatment environment may have pH levels that are unfavorable for microbial activity depending upon site conditions (Kastner et al. 2000; Siegrist et al. 2001). Additionally, permanganate residuals in the source zone or oxygen produced during treatment is likely to maintain oxidative conditions, which prohibit reductive dechlorination of chloroethenes.

### Thermal treatment.

**Figure 4:**
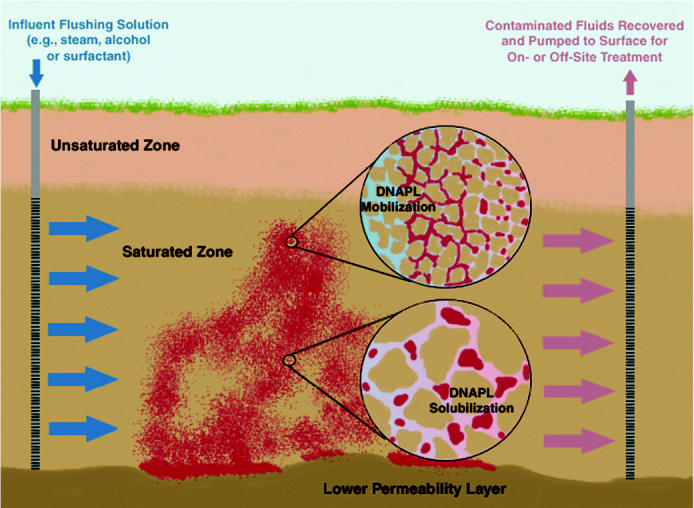
Representation of subsurface flushing technologies in a shallow, relatively homogeneous, unconfined aquifer with a well-defined DNAPL source zone (generalized to include steam, co-solvent, and surfactant). Insets represent DNAPL recovery mechanisms (top, mobilized bank of free product collecting DNAPL ganglia; bottom, reduction in entrapped DNAPL mass through solubilization).

Thermal treatment techniques include steam (or hot water) flooding, resistive heating (e.g., three- or six-phase heating), conductive heating (e.g., thermal blankets), or some combination thereof [for more detailed descriptions of several thermal technologies, see Falta (2000); NRC (1999); Udell (1997)]. Of these technologies, steam flushing is frequently employed for treatment of sites contaminated with NAPL (Figure 4). Laboratory and field tests have demonstrated the robustness of steam flushing (Udell 1997). There are, however, two drawbacks limiting widespread implementation: *a*) energy demands contribute significantly to project costs (Henry et al. 2003) and *b*) the potential for NAPL mobilization (Davis and Heron 1998; Falta 2000). During steam flushing, DNAPL mobilization occurs through a reduction in capillary forces at the condensation front and may become problematic if the recondensed organic liquid phase escapes hydraulic control and contaminates pristine regions of the subsurface. Thus, recent work has focused on designs that reduce the potential for downward migration of DNAPLs during steam flooding (Kaslusky and Udell 2002). Lesser understood impacts of steam treatment include the potential formation of intermediates or byproducts during thermal degradation (Cai and Guengerich 1999; Davis and Heron 1998; Kline et al. 1978; McKinney et al. 1955), and effects of steam and high temperatures on the microbial community (Davis 1998; Richardson et al. 2002).

Long-term monitoring efforts provide limited evidence that microbial activity may rebound after field-scale steam treatment (Smith et al. 1998, 2000). Richardson et al. (2002) found that mesophilic bacterial and archaeal populations survived steam treatment in laboratory studies using soils collected from contaminated sites. In their study microbial activity was only detectable after periods of gradual cooling; elevated temperatures and fast cooling rates resulted in little or no microbial activity. *In situ* rates of cooling are anticipated to be slow enough to allow subsequent microbial rebound (Richardson et al. 2002). Thorough characterization of the sub-surface environment after thermal treatment of DNAPL source zones has yet to be reported, but it is likely that the treated zone immediately after steam or hot water injection will be aerobic, given that air may be injected during treatment for the purposes of contaminant oxidation (Leif et al. 1998) or DNAPL mobility control (Kaslusky and Udell 2002). In contrast, redox potentials measured at a site after electrical resistive heating were found to be consistent with those required for reductive dechlorination (Beyke et al. 2000; Smith et al. 2000). Therefore, additional research is required to determine the effectiveness of employing microbial reductive dechlorination after thermal treatment of DNAPL source zones.

### Co-solvent flushing.

Alcohols have been used as co-solvents to enhance recovery of NAPLs through either solubilization or mobilization (displacement) [Figure 4; for description of the mechanisms and implementation of co-solvent flushing technologies, see Advanced Applied Technology Demonstration Facility (AATDF) (1997); Augustijin et al. (1997); Falta (1998)]. During solubilization, NAPL remains relatively immobile throughout recovery. In contrast, mobilization relies upon reduced capillary forces resulting from a decrease in interfacial tension to facilitate release and displacement of NAPL ganglia, which are recovered as an organic liquid or free product. Mobilization and solubilization are not mutually exclusive processes; co-solvent floods may be designed to favor either mechanism through a detailed understanding of system phase behavior (Brandes and Farley 1993; Falta 1998). Although selection of alcohols to promote partitioning leading to reductions in the density difference between phases (e.g., Lunn and Kueper 1999) can mitigate downward migration of DNAPL, field implementation of mobilization co-solvent floods have been limited to the treatment of light NAPL source zones (Falta et al. 1999). Other field tests employing the use of co-solvents focused on enhanced removal through solubilization (Jawitz et al. 2000; Rao et al. 1997). Use of high concentrations of alcohols (> 70%) in co-solvent flushing may result in gravity override (bypassing) and reduced source-zone bioactivity. Gravity override can be limited with careful design of injection systems to counter buoyancy forces (Jawitz et al. 2000). Although flushing with concentrated alcohol solutions may negatively affect microbial activity, long-term monitoring results (> 3 years) from a site where co-solvent flushing was employed suggest that general bioactivity may rebound as alcohol concentrations decrease (Annable 2003; Mravik et al. 2003). It is unclear, however, how the populations critical to reductive dechlorination respond to alcohol flushing. In general, if harmful impacts on the microbial community can be avoided or are shown to be less disruptive than currently perceived, the addition of short-chain alcohols such as ethanol may prove to be a feasible method for stimulating posttreatment reductive dechlorination.

### Surfactant-enhanced aquifer remediation.

SEAR refers to *in situ* flushing technologies that use surfactants to overcome many of the limitations experienced during pump-and-treat remediation of DNAPL source zones (Figure 4; for mechanistic and practicable descriptions of SEAR, see, e.g., AATDF (1997); Jafvert (1996); Pennell and Abriola 1997)]. Generally, surfactants are molecules that preferentially accumulate at surfaces or interfaces based upon their amphiphilic molecular structure. Both anionic and nonionic surfactants have demonstrated potential for use in NAPL-contaminated aquifer remediation (Baran et al. 1994; Dwarakanath et al. 1999; Pennell et al. 1993; Shiau et al. 1994). SEAR technologies are similar to co-solvent flushing in that the general mechanisms of source-zone mass removal are solubilization and mobilization (Figure 4 inset). As is the case with most aggressive remediation approaches, SEAR leverages greater upfront capital expenditures than traditional pump-and-treat remediation for higher efficiency. More than 90% recovery of contaminant mass has been demonstrated within DNAPL source zones in short time periods at the field scale (Abriola et al. 2005; Londergan et al. 2001; Ramsburg et al. 2005). The efficiency of SEAR makes it an attractive alternative to pump-and-treat remediation where hydraulic control allows for near complete capture of injected surfactant. One drawback to the use of surfactant solutions designed for high contaminant solubilization is the possibility of downward migration of the relatively dense solubilized plume or mobilized free-product DNAPL before recovery. Plume plunging behavior, however, may be mitigated through the addition of alcohols to the surfactant solution (Kostarelos et al. 1998) and careful design of the hydraulic flow regime/control system (Abriola et al. 2005). Concerns over downward migration of mobilized DNAPL may be alleviated by using SEAR technologies that reduce DNAPL density *in situ* before mobilization (Ramsburg et al. 2003; Ramsburg and Pennell 2002; Yan et al. 2003)

Use of readily biodegradable, food-grade surfactants minimizes concerns over the fate of unrecovered surfactant, yet the effect of such surfactants on microbial populations responsible for reductive dechlorination within the swept zone is only now beginning to be explored. Although most anionic and nonionic surfactants considered for application are completely degradable under aerobic conditions (Swisher 1987), degradation of alkylphenol ethoxylates (e.g., Triton X-100) has been shown to generate products (e.g., alkylphenols) that are persistent, toxic, and estrogenic (e.g., Ahel et al. 1994a, 1994b; Stephanou and Geiger 1982; White et al. 1994). Residual levels of readily degradable, food-grade surfactants, however, will likely promote the establishment of anaerobiosis, potentially facilitating conditions conducive for reductive dechlorination.

Application of biodegradable anionic surfactants at field sites has typically been accompanied by high concentrations of 2-propanol (~40 g/L) and sodium chloride (as high as 7 g/L) to increase contaminant solubilization capacities > 60 g/L (e.g., Brown et al. 1999). Thus, posttreatment conditions will likely have elevated concentrations of anionic surfactant, alcohol, and sodium chloride, which could inhibit or prevent microbial activity. Unfortunately, no long-term monitoring results have been reported, limiting the understanding of microbial activity after treatment with these formulations. In contrast, long-term monitoring results from a field test conducted using a biodegradable, food-grade, nonionic surfactant (without alcohol or salt addition) indicate that surfactant degradation stimulated microbial activity within the treated source zone (Ramsburg et al. 2004).

### Implications for coupling physical–chemical treatment with microbial reductive dechlorination.

Existing evidence suggests certain physical–chemical source-zone treatment technologies are more promising for the stimulation of microbial activity as a post-treatment polishing step. Although air sparging, chemical oxidation, and steam flooding may generate an aerobic environment suitable for subsequent metabolic or co-metabolic oxidation, SEAR and co-solvent flushing appear to be the most promising physical–chemical treatments for integration with the microbial reductive dechlorination process. Note that in this assessment, the possibility that DNAPL contaminant distributions resulting from aggressive treatment may be technology specific has not been considered because of the scarcity of data. Residual alcohol or surfactant solutions contribute to oxygen depletion and establishment of anaerobic conditions after aggressive treatment. Further, residual flushing solution may serve as a source of reducing equivalents and stimulate the reductive dechlorination process. Although other technologies may eventually be successfully integrated with posttreatment microbial reductive dechlorination, SEAR seems particularly applicable because of limited toxicity on the microbial community, the establishment of reducing conditions, and the release of reducing equivalents for stimulation of the reductive dechlorination process. Thus, the ultimate fate of the residual surfactant solution and its effect on the dechlorinating population must be considered.

Although microbial degradation of surfactants in aerobic environments is well documented (Swisher 1987), it is uncertain how surfactants typically selected for SEAR are degraded in anaerobic environments. Linear alcohol ethoxylates are degraded to fermentable substrates under anaerobic conditions (Huber et al. 2000), and the degradation of nonionic surfactant has been reported under methanogenic conditions (Yeh et al. 1999). It is therefore, likely that fermentation of unrecovered surfactant will serve as an indirect source of reducing equivalents by producing hydrogen and organic acids, whose slow anaerobic oxidation will generate additional hydrogen to support the chlororespiring populations.

The residual surfactant concentrations, however, may also alter the bioavailability of a contaminant (Colores et al. 2000; Pennell et al. 2001; Rouse et al. 1994). Yeh et al. (1999) investigated the bioavailability of hexachlorobenzene (HCB) in the presence of non-ionic, ethoxylated sorbitan surfactants (i.e., Tween series) in a methanogenic mixed culture obtained from contaminated sediment. At low surfactant concentrations (< 10 mg/L) there was no apparent change in rate or extent of HCB dechlorination. At a surfactant concentrations above the critical micelle concentration (CMC), enhanced HCB dissolution occurred, and although dechlorination rates decreased, the dechlorination end point remained unchanged. Complete inhibition of reductive dechlorination was observed at a surfactant concentration of 1,000 mg/L. However, Yeh et al. (1999) hypothesized that the observed inhibition was likely due to toxic effects of high surfactant concentrations rather than micellar sequestration of HCB. These results are supported by a recent study using a PCE dechlorinating consortium and a matrix of anionic, nonionic, and cationic surfactants (McGuire and Hughes 2003). McGuire and Hughes (2003) observed that the nonionic surfactant Tween 80 [polyoxyethylene (20) sorbitan monooleate] exhibited the least impact on dechlorination (both rate and extent) and thus speculated that the number of ethylene oxide groups present on the surfactant molecule affects surfactant toxicity. In fact, Bury and Miller (1993) and Guha et al. (1998) demonstrated that contaminants (in these studies nonchlorinated hydrocarbons) sequestered in the micellar phase may remain bioavailable. The response of the dechlorinating microbial community to surfactants is poorly understood, and future research should explore possible stimulatory or inhibitory effects in a heterogeneous environment where local surfactant concentrations may be well above the CMC.

## Mathematical Assessment

Although microbial reduction of PCE in DNAPL source zones may be feasible, the relatively low dissolution enhancement factors (3- to 6-fold) reported imply that source longevity would still be measured in multiple decades. Alternatively, if uncertainties in the source zone microbial environment after physical–chemical treatment can be overcome, multiple order-of-magnitude reductions in source-zone mass removal obtained via active physical–chemical treatment might be combined with posttreatment biopolishing to substantially reduce source longevity. Ultimately, it may be possible to devise a posttreatment source-zone strategy that minimizes operations and maintenance efforts while still meeting regulatory standards at down-gradient points of compliance.

**Figure 5:**
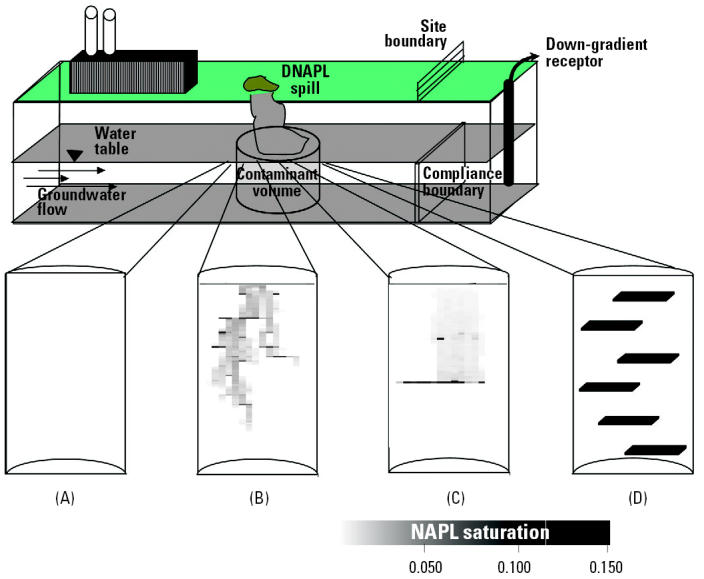
Depiction of DNAPL source-zone conceptual models used in the example calculations: (*A*) IGP ratio, (*B*) HGP ratio, (*C*) LGP ratio, and (*D*) ZGP ratio. All control volumes are the same size and contain equal amounts of PCE-DNAPL.

The potential benefits of tailoring physical–chemical treatments to stimulate microbial reductive dechlorination may be illustrated through a straightforward mathematical modeling analysis that compares source longevity for four hypothetical DNAPL source-zone scenarios (Figure 5) under three management strategies: *a*) natural gradient dissolution (natural dissolution), *b*) enhanced reductive dechlorination (source-zone bioremediation), and *c*) physical–chemical treatment followed by source-zone biopolishing (SEAR plus enhanced reductive dechlorination). The four hypothetical field scenarios were selected to span the range of behavior that may be expected in the field and are characterized by a ganglia-to-pool (GTP) ratio, which is a measure of the distribution of mass between low saturation ganglia regions and high saturation pool regions in the source zone. The formation properties, spill scenario, and SEAR characteristics were taken from a recent numerical modeling study that was based on a pilot-scale SEAR demonstration at the Bachman Road site in Oscoda, Michigan (Abriola et al. 2005; Lemke and Abriola 2003; Lemke et al. 2004). These properties are summarized in Table 2. Scenario 1 assumes NAPL is entrapped as residual globules and ganglia at a uniform saturation throughout the source zone (Figure 5A). This scenario has an infinite GTP ratio (IGP) and would be characteristic of an ideal site that had perfectly uniform hydraulic properties and where DNAPL was released over a reasonably wide area. Cleanup of this site is modeled using a simplified hydraulic approach (Brusseau 1996), which is based on mass-balance calculations. Scenario 2 is perhaps more realistic. It is representative of a situation with the NAPL entrapped as residual ganglia (Figure 5B), although some pooling has occurred because of permeability contrasts [high GTP ratio (HGP), GTP > 1.0]. This DNAPL saturation distribution was generated following the methods outlined by Lemke and Abriola (2003) and Lemke et al. (2004). Using this methodology, the release of NAPL into a nonuniform permeability field is simulated using an laboratory-validated numerical multiphase simulator (MVALOR; Dekker and Abriola 2000; Lemke et al. 2004; Rathfelder et al. 2001). Natural dissolution or SEAR is then simulated using a separate numerical simulator (MISER) that has been used to accurately simulate SEAR in laboratory experiments (Rathfelder et al. 2000, 2001) and was used in the design of a recent SEAR pilot-scale test (Abriola et al. 2005). Scenario 3 was also generated using this same methodology (Figure 5C). Here, however, formation properties were configured so that the resultant saturation distribution was dominated by pools [low GTP ratio (LGP), GTP < 1.0; for details, see Lemke et al. (2004)]. Scenario 4 assumes all mass is immobilized in six idealized, rectangular, fully saturated (*S_n_* = 1) pools with no ganglia remaining (Figure 5D). This scenario is an extreme case where the GTP ratio is equal to zero (ZGP). Cleanup in this scenario was modeled using an analytical solution to the two-dimensional advection-dispersion equation following the methods of Johnson and Pankow (1992). It should be noted that, in contrast to the HGP and LGP scenarios (1 and 4), which result from the use of numerical models that incorporate more of the physics of the problem (e.g., hysteretic DNAPL migration, nonuniform flow, rate-limited dissolution), the IGP and ZGP scenarios are nonphysical, idealized end-members intended to bracket behavior that may be observed in the field. Although the distribution of mass in the source zone is different in each of the four scenarios, the amounts of mass in the source zone, the source-zone (i.e., domain) volume, the aqueous-phase contaminant solubility during a given process (i.e., SEAR or natural gradient dissolution), and the average hydraulic flux through the source zone are identical.

The source longevity in scenarios 1–4 using each of the three remediation strategies was arbitrarily defined as the time when 99.9% NAPL was removed from the source zone. The second and third management strategies, source-zone bioremediation and SEAR plus biopolishing, used a simplified bioenhancement factor taken from the literature to quantify the improvement in dissolution because of microbially mediated aqueous-phase degradation. Reductive dechlorination enhanced-dissolution factors ranging from 3- to 6-fold have been reported (Cope and Hughes 2001; Yang and McCarty 2002). For this simplified example, an enhancement factor of 5 was assumed. This enhancement factor was reported in column studies in which NAPL ganglia were uniformly distributed, chlororespirers were present and active, and there were no limitations on microbial growth (Cope and Hughes 2001; Yang and McCarty 2000). It is unlikely that these conditions could be obtained at real sites, and thus, the enhancement factor of 5 is likely optimistic. However, in an effort to determine the benefits of aggressive mass removal before source-zone biopolishing (management strategy 3) versus bioremediation alone, favorable source-zone bioremediation (management strategy 2) was assumed.

**Figure 6:**
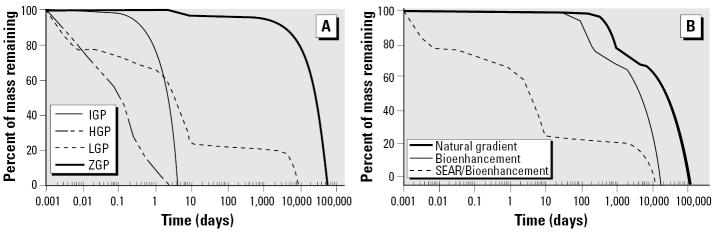
Percent DNAPL mass remaining as a function of time for (*A*) SEAR followed by bioenhancement in all four scenarios and (*B*) three alternative remediation strategies in LGP scenario.

Calculated values of source longevity for each of the three management strategies for all four scenarios are reported in Table 3, and percent mass removal as a function of time is presented in Figure 6. As might be expected, source longevity for scenario 1 (IGP) and scenario 4 (ZGP) tends to bracket the cleanup behavior of the more complex scenarios (HGP and LGP). Application of physical–chemical source-zone treatment (a 10-day surfactant flush of 4% Tween 80) before biopolishing is shown to reduce the source longevity regardless of scenario conditions. The magnitude of this reduction, however, depends on the level of pooling in the NAPL source zone (Figure 6A). If, for example, the LGP scenario is assumed to be representative of a typical small-scale site, the 10-day SEAR followed by biopolishing will result in a 53 and 91% decrease in source longevity, in comparison with results of source-zone reductive dechlorination alone and natural dissolution conditions, respectively (Figure 6B). In this scenario, conducting SEAR operations for an additional 15 days (25 days total) would result in removal of 98.5% of the DNAPL mass, thereby reducing source longevity to 4 years. Thus, results presented in Table 3 and Figure 6 suggest that physical–chemical treatment followed by enhanced microbial activity could greatly reduce source longevity and associated long-term risk.

## Bachman and Sages

The co-solvent flood at the former Sages dry cleaning facility (Jacksonville, Florida) and the Bachman Road SEAR site (Oscoda, Michigan) serve as documented case studies where field evidence supports the conclusion that physical–chemical source-zone removal may be coupled with reductive dechlorination. A comparison between observations at the Sages and Bachman sites is shown in Table 4. It is important to recognize that these posttreatment monitoring data provide only a snapshot of conditions (at 1,280 days for Sages and 450 days for Bachman) in a transient environment. Although the evolutions of the conditions at the Sages and Bachman sites are described in more detail in Mravik et al. (2003) and Ramsburg et al. (2004), respectively, we provide a summary below to facilitate analysis of the observed stimulation of microbial reductive dechlorination after physical–chemical treatment.

At the Sages site, 34,000 L of a solution consisting of 95% (vol) ethanol and 5% (vol) water were flushed through a DNAPL source zone over a period of 3.5 days followed by a 4.5-day water flood used to recover injected fluids (Jawitz et al. 2000). This co-solvent flood was successful in removing 43 L of PCE-DNAPL from the subsurface, and extraction well data indicate 92% of the ethanol introduced during the flush was recovered (Jawitz et al. 2000). Posttreatment characterization conducted approximately 1 month after the cessation of flushing activities indicated that DNAPL remained after treatment (Sillan 1999) and that the average PCE and ethanol concentrations in the extraction wells were ~120 μM and ~ 230 mM, respectively (Mravik et al. 2003). Results from longer-term sampling at the Sages site indicate that PCE concentrations within the source zone rebounded to pretreatment levels approximately 150 days after treatment and that ethanol concentrations remained in excess of 160 mM for approximately 350 days (Mravik et al. 2003). Although ethanol toxicity remains a concern, elevated concentrations of hydrogen and acetate in the treated zone suggest microbial activity (Mravik et al. 2003). Soil samples taken from a core collected down-gradient of the Sages treated zone tested positive when analyzed via nested polymerase chain reaction with *Dehalococcoides*-targeted primers (Mravik et al. 2003). Additionally, microcosm studies with aquifer material from the Sages site indicate that sulfate-reducing and methanogenic populations rebounded after exposure to elevated concentrations of ethanol (Ramakrishnan et al. 2005). Although the survival and activity of dechlorinating populations within the treated zone have not been demonstrated to date, observations of significant *cis*-DCE production (up to 242 μM) at monitoring points located within the treated zone are indicative of microbial reductive dechlorination.

At the Bachman Road site, a pilot-scale field demonstration of SEAR was conducted to remove PCE-DNAPL from beneath a former dry cleaning facility. For this source-zone treatment, 68,400 L of an aqueous solution containing 6% (weight) Tween 80 were introduced over a period of 10 days, with 2 additional days of active water flooding (Abriola et al. 2005; Ramsburg et al. 2005). Approximately 95% of the injected surfactant was recovered along with > 19 L of PCE. Posttreatment site monitoring indicates that PCE concentrations were reduced by two orders of magnitude from pretreatment levels at many locations within the treated zone and, in contrast to the Sages site, did not rebound after 450 days (Ramsburg et al. 2004). Surfactant concentrations decreased steadily over time, and after 270 days, surfactant was not detectable at most sampling points within the treated zone (12 μM detection limit).

Before the SEAR treatment, substantial reductive dechlorination had not occurred in the source zone. However, significant concentrations of PCE degradation products were measured within the treated zone 270 days after treatment (Table 4). Acetate and formate, likely products of Tween 80 fermentation, were observed at levels as high as 4,600 μM and are indicative of anaerobic microbial degradation of the surfactant (Ramsburg et al. 2004). Organic acids are known to support reductively dechlorinating populations present in the Bachman aquifer (He et al. 2002, 2003a, 2003b; Sung et al. 2003), and PCE–to–*cis*-DCE transformation within the treated source zone is consistent with laboratory microcosm studies conducted with aquifer material from the Bachman Road site (He et al. 2002). VC, however, was detected at only 3 of 26 sampling locations within the source zone. The apparent accumulation of *cis*-DCE at most observation locations may indicate that PCE–to–*cis*-DCE degrading organisms are predominating within the treated zone.

These two examples from field sites suggest that physical–chemical source-zone treatments are capable of stimulating organisms responsible for degrading residual level contaminants. At these sites, data support the conclusion that ethanol and Tween 80 were metabolized by active microbial communities, resulting in an increased production of hydrogen and acetate. The availability of these electron donors, in turn, promoted reductive dechlorination activity. Although such enhanced bioactivity within source zones may occur at sites contaminated on much larger scales (e.g., Hill Air Force Base; Londergan et al. 2001), it is important to recognize that sites such as Sages and Bachman are representative of numerous small-scale chloroethene source zones existing in communities across the United States (e.g., State Coalition for Remediation of Drycleaners 2004). These smaller sites not only are significant sources of dissolved phase contamination but are often more problematic because *a*) they typically occur in proximity to areas of higher population, increasing risk and limiting hydraulic isolation (i.e., containment) options, and *b*) the relatively low NAPL saturations and smaller treated volumes at these sites increase treatment costs as quantified by conventional metrics (dollars per cubic meter of treated soil or dollars per liter of NAPL recovered). Higher costs per volume (treated soil or NAPL) result from a threshold cost associated with establishing a treatment system regardless of site size. Many innovative source-zone technologies offer efficient mass removal at the expense of greater, upfront capital expenditures (Rao et al. 2002). Decreased source longevity resulting from aggressive treatment, however, results in lower operational and maintenance costs making many innovative approaches economically viable when compared against long-term pump-and-treat remediation (e.g., Ramsburg and Pennell 2001). A staged treatment approach that employs microbial reductive dechlorination after aggressive mass removal may thus provide a cost-effective option for reduction of both source longevity and risk.

The need for integrating treatment technologies for groundwater cleanup has become more apparent (Jackson 2003; Rao et al. 2002) since first being advocated by the NRC’s Committee on Ground Water Cleanup Alternatives (NRC 1994). Thorough site characterization is critical for design of any treatment train remedy (Jackson 2003). Site-specific tailoring of physical–chemical treatment for stimulation of posttreatment bioactivity must be based upon an accurate understanding of the location and extent of DNAPL, as well as hydrogeology and pretreatment microbial parameters. Co-solvent and surfactant flushing are very promising approaches because they can be tailored to enhance posttreatment reductive dechlorination. It should be noted, however, that ISCO may provide another means of polishing of residual-level contamination subsequent to other source-zone remediation technologies. Additionally, ISCO may be an attractive follow-on treatment alternative at sites where characterization efforts demonstrate that dechlorinating populations cannot be readily stimulated or augmented.

## Conclusions

Taken in total, literature data, example calculations, and case studies presented above support a position of cautious optimism regarding the potential of combined physical–chemical/reductive dechlorination remedial methods for the effective treatment of chlorinated solvent source zones. The literature review, however, suggests a number of areas requiring further investigation before the performance of such methods can be fully assessed and optimized. Given the number of remediation sites at which natural attenuation of chlorinated solvents has been documented (Wiedemeier et al. 1999), and the knowledge that many of the flushing solutions themselves stimulate bioactivity in laboratory tests, one would anticipate that stimulation of indigenous microorganisms in a source zone after physical–chemical treatment would be common. Therefore, the lack of widespread evidence for bioremediation after physical–chemical treatment indicates either that microbial activity is occurring but lacks documentation (e.g., the indicators of bioremediation are not monitored) or that the posttreatment environment does not favor microbial activity. It is important that future field demonstrations of source-zone flushing technologies are designed to systematically investigate *a*) the source-zone (dechlorinating) microbial community, before, during, and after the treatment process, and *b*) contaminant and transformation product concentration distributions after treatment. Indeed, to date, most field observations of enhanced reductive dechlorination in treated source zones have been fortuitous, with little thought devoted to microbial processes in the initial design and implementation of the treatment monitoring scheme. Specific culture-dependent (e.g., microcosms) and culture-independent (nucleic acid-based) tools for assessment of the microbial community are now available for this characterization effort (He et al. 2003a, 2003b; Hendrickson et al. 2002; Löffler et al. 2000; Morse et al. 1998).

Future field demonstrations may also be enhanced through exploitation of results obtained from microbial laboratory investigations. Laboratory-scale studies conducted under conditions representative of a source-zone environment (i.e., in the presence of organic liquid) provide heuristic, as well as quantitative, guidance for implementation of posttreatment bioremediation. Substrate amendment strategies that favor chlororespiring populations by maintaining a low concentration of hydrogen may be adapted from the laboratory to the field. However, additional work will be required to explore the effect of unrecovered flushing solutions (e.g., alcohol or surfactant) typical of a posttreatment source-zone environment on the metabolism of chlorinated NAPLs by chlororespiring organisms. The discovery of numerous dechlorinating populations capable of converting PCE to *cis*-DCE and recognition of the importance of *Dehalococcoides* populations in the transformation of chloroethenes to ethene will likely improve future bioaugmentation strategies and further enhance posttreatment biopolishing. Although enhanced NAPL dissolution by partially dechlorinating populations has been demonstrated, it remains to be seen if complete detoxification (e.g., ethene formation) in source zones is feasible.

## Figures and Tables

**Figure 1:**
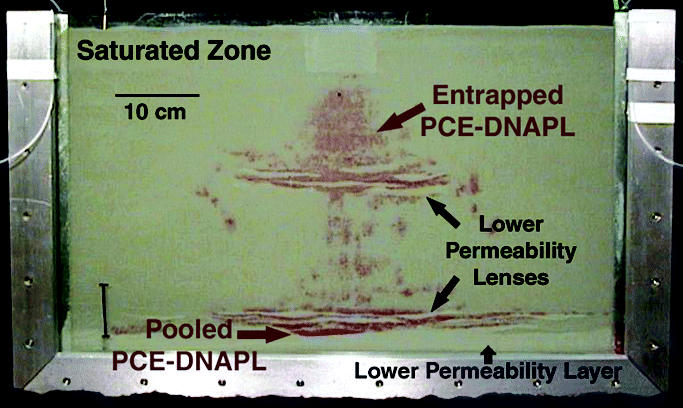
Representative photograph from laboratory-scale (63.5 cm length × 38 cm height × 1.4 cm thickness) infiltration and entrapment PCE-DNAPL (dyed red with 10^−4^ M Oil Red-O for visualization).

**Table 1:** Summary of anaerobic and aerobic processes involved in dechlorination/degradation of chlorinated ethenes.

	Oxidation processes		Co-metabolic processes		
	Anaerobic (energy yielding)	Aerobic (oxygen dependent, energy yielding)	Anaerobic reduction	Aerobic oxidation	Chlororespiration: anaerobic (energy-yielding reduction)
Metabolic group(s)	Fe(III) reducers Mn(IV) reducers Humic acid reducers	*Mycobacterium* spp. *Nocardioides* spp. *Pseudomonas* spp. *Polaromonas* sp.	Sulfidogens Methanogens Acetogens	Organisms with broad range oxygenases	Chlororespirers
Relative dechlorination/degradation rates	Unknown	++	+	++*^a^*	++++
Frequency of active organisms in nature	Unknown	VC oxidizers widely distributed in aerobic environments	High in anaerobic environments	High in aerobic environments	Not rare in anaerobic environments
Favorable site conditions	Fe(III) reducing Mn(IV) reducing	Aerobic	Anaerobic, not e^−^ donor or e^−^ acceptor limited	Aerobic, primary substrate present	Anaerobic, appropriate e^−^ donor present, no interfering TEAPs

Abbreviations: e^−^, electron; TEAP, terminal electron-accepting process; +, slow rate; ++, moderate rate; +++, fast rate; ++++, very fast rate.

a Dechlorination rates are often not sustained because of accumulation of toxic intermediates.

**Table 2:** Parameters and values used in example calculations.

Parameter	Value	Units	Reference
PCE spill volume	0.096	m^3^	Lemke et al. 2004
Spill radius (*r*)	0.797	m	Lemke et al. 2004
Spill depth (*h*)	8.315	m	Lemke et al. 2004
Average NAPL Saturation (*S*_o_^Avg^)	0.017	—	Lemke et al. 2004
Porosity (*n*)	0.36	—	Lemke et al. 2004
PCE density (ρ_PCE_)	1.623 × 10^6^	g/m^3^	Verschueren 1983
Rate-limited aqueous-phase PCE concentration (*C*_aq_^PCE^)	30*^a^*	g/m^3^	Abriola et al. 2005
Length of surfactant flush	10	day	Abriola et al. 2005
Bioenhanced dissolution factor	5*^a^*	—	Carr et al. 2000 Carr et al. 2000 Cope and Hughes 2001 Yang and McCarty 2002
Apparent PCE concentration during SEAR (*C*)	5.4 × 10^3^	g/m^3^	Ramsburg et al. 2005
Groundwater velocity (*V*_d_)	0.032	m/day	Lemke et al. 2004
Groundwater velocity during SEAR	0.514	m/day	Abriola et al. 2005 Ramsburg et al. 2005
Pore volume	5.9	m^3^	Calculated
Pool length (*L*_p_)	1	m	Calculated
Pool depth	0.016	m	Calculated
Number of independent pools	6	—	Calculated
Vertical dispersivity (α_v_)	2.3 × 10^−4^	m	Johnson and Pankow 1992
Aqueous solubility of PCE	150	g/m^3^	Verschueren 1983
Equilibrium solubility of PCE in surfactant solution	26,880*^b^*	g/m^3^	Taylor et al. 2001
PCE bulk aqueous phase diffusion coefficient (*D*_aq_^PCE^)	5.7 × 10^−5^	m^2^/day	Dekker and Abriola 2000

a Assumed based upon range of reported values.

b From reported weight solubilization ratio of 0.672 g of PCE per gram of surfactant (4% Tween 80 solution).

**Table 3:** Calculated source longevities (years).

Scenario	Natural gradient dissolution	Source-zone bioremediation*^a^*	SEAR + biopolishing
1. Infinite ganglia-to-pool ratio	36	7	0.01*^b^*
2. High ganglia-to-pool ratio	54	11	0.01*^b^*
3. Low ganglia-to-pool ratio	245	50	24
4. Zero ganglia-to-pool ratio	817	163	157

a Source-zone bioremediation calculations assume active chlororespiring organisms are present in sufficient numbers and no nutrient or substrate limitations for duration of treatment.

b Ten-day SEAR (4% Tween) alone was sufficient for 99.9% removal of PCE-DNAPL mass.

**Table 4:** Comparison of key site parameters and monitoring data.

Site	Sages	Reference	Bachman	Reference
Site characteristics				
Location	Jacksonville, FL	Jawitz et al. 2000	Oscoda, MI	Abriola et al. 2005
Former site use	Dry cleaner	Jawitz et al. 2000	Dry cleaner	Abriola et al. 2005
Primary contaminant	PCE	Jawitz et al. 2000	PCE	Abriola et al. 2005
Depth to groundwater*^a^*	2.0–2.6 m	Jawitz et al. 2000	2.4–3.0 m	Abriola et al. 2005
Depth to confining unit	10 m	Jawitz et al. 2000	7.6 m	Abriola et al. 2005
Range of hydraulic conductivity*^b^*	3–6 m/day	Jawitz et al. 2000	1–48 m/day	Abriola et al. 2005
Soil classification	Fine grain sands	Jawitz et al. 2000	Medium to fine grain sands	Abriola et al. 2005
Areal extent of treated zone	7.3 × 2.7 m	Jawitz et al. 2000	4.3 × 6.7 m	Abriola et al. 2005
Estimated overall NAPL saturation	0.004	Jawitz et al. 2000	0.0004	Ramsburg et al. 2005
Maximum observed PCE aqueous concentration preceding treatment	710 μM	Jawitz et al. 2000	600 μM	Ramsburg et al. 2005
Management strategy				
Treatment	Co-solvent	Jawitz et al. 2000	SEAR	Abriola et al. 2005
Flushing solution	95% (vol) ethanol	Jawitz et al. 2000	6% (wt) Tween 80	Ramsburg et al. 2005
Volume of solution injected	34 kL	Jawitz et al. 2000	68 kL	Ramsburg et al. 2005
Duration of injection	3 day + 4.5 day waterflood	Jawitz et al. 2000	10 day + 2 day waterflood	Ramsburg et al. 2005
Recovery of active ingredient	92% (ethanol)	Jawitz et al. 2000	95% (Tween 80)	Ramsburg et al. 2005
PCE mass recovery	43 L	Jawitz et al. 2000	19 L	Ramsburg et al. 2005
Unrecovered active ingredient	2,000 kg (45 kmol)	Mravik et al. 2003	225 kg (0.17 kmol)	Ramsburg et al. 2005
Total cost	$440,000	Sillan 1999	$365,900	Ramsburg et al. 2005
Monitoring				
Posttreatment monitoring period	1,280 day	Mravik et al. 2003	450 day	Ramsburg et al. 2004
Range and (median) of concentrations within treated zone at last reported monitoring*^c^*				
PCE	50–150 (100) μM	Mravik et al. 2003	0.11–36 (0.38) μM	Ramsburg et al. 2004
TCE	10–30 (20) μM	Mravik et al. 2003	0.01–91 (0.09) μM	Ramsburg et al. 2004
*cis*-DCE	36–242 *^d^* (150) μM	Mravik et al. 2003	0.17–1,032 (2.2) μM	Ramsburg et al. 2004
VC	0.07–13 *^d^* (2.0) μM	Mravik et al. 2003	0.02–6.6 (0.02) μM	Ramsburg et al. 2004
Ethene	0.04–0.43 *^d^* (0.20) μM	Mravik et al. 2003	Not measured	Ramsburg et al. 2004
Acetate	200–600 (400) μM	Mravik et al. 2003	100–4,600 (100) μM	Ramsburg et al. 2004

a Varies seasonally.

b Range due to spatial variability within source zone.

c Values for the Sages site are estimated from kriged contours.

d Actual range [i.e., non-kriged range reported in Mravik et al. (2003)].
